# Mechanical and Thermal Properties of DCPDA-Modified THEICTA/DMAA Photocurable Resins for LCD 3D Printing

**DOI:** 10.3390/ma19132845

**Published:** 2026-07-03

**Authors:** Ruiying Chen, Jingwei He

**Affiliations:** 1 School of Materials Science and Engineering, South China University of Technology, Guangzhou 510641, China; 202421020626@mail.scut.edu.cn; 2 Key Lab of Guangdong High Property and Functional Macromolecular Materials, School of Materials Science and Engineering, South China University of Technology, Guangzhou 510640, China

**Keywords:** 3D printing, photocurable resins, high strength, heat resistance

## Abstract

Conventional photocurable resins suffer from low mechanical strength and poor thermal stability, which restrict their broader application in photopolymerization-based 3D printing technologies. Strategies such as inorganic fillers or interpenetrating polymer networks (IPNs) improve performance but often lead to high viscosity or long post-curing times. In this work, a high-performance photocurable resin system based on tris(2-hydroxyethyl) isocyanurate triacrylate (THEICTA) and N, N-dimethylacrylamide (DMAA) was developed, achieving low viscosity and direct room-temperature 3D printability. Partial substitution of DMAA with Tricyclodecanedimethanol diacrylate (DCPDA) further enhanced its mechanical and thermal properties. The optimized resin exhibited a tensile strength of 80.9 MPa, a flexural strength of 166.1 MPa, a glass transition temperature of 176.2 °C, and a low viscosity of 78.7 mPa·s with shrinkage below 10%. The heat deflection temperatures reached 168.1 °C under 1.80 MPa and 187.3 °C under 0.45 MPa. This study provides an effective strategy for developing high-strength and heat-resistant photocurable resins suitable for efficient room-temperature 3D printing applications.

## 1. Introduction

Three-dimensional (3D) printing, also known as additive manufacturing (AM), is an advanced fabrication technology that constructs objects through the layer-by-layer deposition of materials according to digital models [1,2]. Compared with conventional manufacturing techniques, 3D printing offers greater design freedom and enables the fabrication of complex geometries and customized architectures with high precision [3]. These advantages have led to its widespread application in rapid prototyping, microfluidics, tissue engineering, electronics, dentistry, and surgical modeling [4,5]. Consequently, 3D printing has been increasingly explored for the fabrication of advanced functional polymer composites.

Photopolymerization-based 3D printing represents one of the most established additive manufacturing technologies. Among various additive manufacturing processes, vat photopolymerization technologies, including stereolithography (SLA), digital light processing (DLP), and liquid-crystal display (LCD) printing, have attracted extensive research attention due to their high fabrication accuracy, excellent resolution, superior surface finish, and rapid processing capability. This technology utilizes light irradiation at a specific wavelength to initiate polymerization of reactive groups in liquid photosensitive resins, thereby forming three-dimensional crosslinked polymer networks [6,7]. The resin formulation plays a critical role in determining printing behavior, curing characteristics, and the final properties of printed objects. Photocurable resins typically consist of reactive monomers or oligomers, photoinitiators, reactive diluents, and functional additives [8]. With the increasing adoption of vat photopolymerization-based 3D printing in engineering applications, the limitations of current photopolymer materials have become increasingly apparent [9,10,11]. Although many commercial photopolymer resins exhibit excellent printability, their mechanical strength and thermal resistance remain relatively limited, with tensile strengths commonly below 60 MPa and glass transition temperatures (T g) generally lower than 140 °C, restricting their application in demanding engineering fields. Therefore, the development of engineering-grade photocurable resins combining excellent printability, high mechanical performance, and superior thermal stability has become an important research focus.

The integration of organic and inorganic components has been considered an effective strategy for developing materials with enhanced multifunctional properties [10]. Previous studies have demonstrated that the incorporation of inorganic micro-/nanofillers, including SiO_2_ [12], Al_2_O_3_ [13], and graphene [14], can effectively improve the fracture resistance of polymer composites. After surface modification, these fillers can interact physically or chemically with the photosensitive resin matrix, thereby suppressing crack propagation and improving energy dissipation. However, the incorporation of inorganic fillers often leads to a significant increase in the viscosity of photocurable resin formulations, which negatively affects their processability and printability [15]. For vat photopolymerization-based 3D printing, resin systems generally require a relatively low viscosity, typically below 1000 mPa·s, to ensure stable printing performance and satisfactory fabrication efficiency [16].

Compared with filler reinforcement, constructing highly rigid photocurable networks through molecular design has emerged as an effective strategy for enhancing the mechanical and thermal performance of photopolymerized materials. In recent years, considerable efforts have been devoted to incorporating rigid structural moieties, such as bismaleimide (BMI), polyimide (PI), and isosorbide derivatives, into photocurable resin systems. Hua et al. synthesized a photocurable BMI resin via Michael addition and subsequent grafting with glycidyl methacrylate, achieving a tensile strength of 72.6 MPa and a T g of 155 °C in SLA-printed parts [17]. Guo et al. developed a polyimide-based photosensitive resin for DLP printing with significantly improved thermal stability [18]. Chu et al. reported an isosorbide methacrylate resin exhibiting a T g above 220 °C [19]. These results highlight the important role of rigid structural units in enhancing the thermomechanical performance of photocurable networks. However, the superior performance of such systems is often achieved through complex monomer synthesis and multistep chemical modification, which not only increase material costs and manufacturing complexity but may also hinder their large-scale production and practical application in commercial desktop LCD/DLP printing applications.

In addition to molecular design strategies, the construction of interpenetrating polymer networks (IPNs) has attracted increasing attention as a promising route toward high-performance photocurable materials. IPN systems typically combine photopolymerization with subsequent thermal curing to generate interconnected network structures with enhanced crosslink density and thermal stability [20]. For example, Wang et al. prepared an engineering-grade 3D-printing resin by blending bisphenol-A epoxy resin with a bio-based methacrylate, resulting in a tensile strength of approximately 60 MPa, improved impact resistance, and low dielectric loss [21]. Zhou et al. introduced THEICTA into a cyanate ester system and constructed an IPN structure through sequential photo–thermal curing, obtaining a 3D-printable resin with tensile strength exceeding 100 MPa together with excellent thermal performance [22]. Chen et al. incorporated bismaleimide and an acrylate liquid-crystal resin into a commercial photosensitive formulation, producing a synergistically crosslinked network with a tensile strength of 81.7 MPa and enhanced heat resistance [23]. Fridman et al. reported a sequential IPN system based on multifunctional maleimide and cyanate ester monomers, achieving a T g above 250 °C, high fracture toughness, and low curing shrinkage [24]. Despite these impressive properties, IPN-based systems generally require prolonged high-temperature post-curing and relatively complicated formulation designs, which increase manufacturing time and processing complexity.

In this work, a THEICTA/DMAA-based photocurable resin system was investigated for LCD 3D printing. The effects of the THEICTA/DMAA ratio on resin viscosity, curing behavior, mechanical properties, and thermal performance were systematically evaluated to determine the optimized formulation. Subsequently, DCPDA was introduced as a partial replacement for DMAA to further regulate the network structure and improve the comprehensive performance of the resin. Unlike previously reported high-performance photocurable systems that rely on complex monomer synthesis or IPN construction, this study focuses on optimizing resin composition using commercially available monomers. The optimized resin exhibits a balanced combination of mechanical properties and thermal performance, providing insights into the formulation design of engineering-grade LCD photocurable resins.

## 2. Materials and Methods

### 2.1. Material Preparation

Free radical photoinitiator ethyl (2,4,6-trimethylbenzoyl) phenylphosphinate (TPO-L) and monomers tris(2-hydroxyethyl) isocyanurate triacrylate (THEICTA) were purchased from Shanghai Guangyi Chemical Co., Ltd. (Shanghai, China). Diluted monomer N, N-dimethylacrylamide (DMAA) was obtained from RYOJI (Shanghai, China). Tricyclodecanedimethanol diacrylate (DCPDA) was obtained from Eternal Materials Co., Ltd. (Kaohsiung, Taiwan).

In this study, THEICTA served as the primary structural monomer. DMAA was employed as a reactive diluent to reduce viscosity and improve printability. DCPDA was introduced as a rigid difunctional comonomer to enhance the mechanical and thermal properties of the cured materials. TPO-L served as the photoinitiator, generating free radicals under UV irradiation to initiate the polymerization reaction.

### 2.2. Preparation of 3D Printable Resins and Printed Samples

THEICTA was first heated to 60 °C to obtain a homogeneous melt. Subsequently, DMAA, DCPDA, and TPO-L was added at a fixed concentration of 2 wt.%, which was selected based on previous literature reports [7]. The composition of the 3D printing resin systems listed in Table 1 is summarized. The resulting mixture was magnetically stirred at room temperature for 2 h until a uniform resin system was obtained, which was then used for subsequent LCD 3D printing. The test samples were printed using an LCD printer (Mono 4, Shenzhen Anycubic Technology Co., Ltd., Shenzhen, China) at room temperature. The layer thickness was set to 50 μm, with an exposure time of 5 s per layer and a bottom exposure time of 5 s for the layers attached to the printing platform. The dimensions of the printed specimens were designed according to the corresponding testing standards. After printing, all samples were washed with 95% ethanol to remove residual uncured resin and then dried at room temperature. Subsequently, the samples were post-cured for 15 min using UV post-curing lamps (Wash&Cure 3.0, Shenzhen Anycubic Technology Co., Ltd., Shenzhen, China), followed by thermal post-curing in a vacuum oven at 150 °C for 2 h to promote the formation of a more complete crosslinked network, and enhance the mechanical and thermal properties of the printed specimens. Finally, the printed specimens were naturally cooled to room temperature before characterization.

To optimize the mechanical performance of the material, a resin ratio of 6:4 was identified through systematic screening. DCPDA was selected to replace the original DMAA component. The detailed formulation is summarized in Table 2.

### 2.3. Assessment of Physical and Chemical Properties

The viscosity of the resins was evaluated using a rotational viscometer (HBDV-II+P, Brookfield, Toronto, ON, Canada) equipped with a S02 spindle. Measurements were conducted at 25 °C with a rotational speed of 160 rpm. Each resin formulation was tested three times, and the average value was reported.

The degree of conversion (DC) of the photocured resins was determined by attenuated total reflection Fourier transform infrared spectroscopy (ATR-FTIR). The spectra were recorded at room temperature in the range of 4000–500 cm^−1^ with a resolution of 4 cm^−1^ and 32 scans. The uncured resin was first analyzed, followed by the characterization of cured samples (15 mm × 15 mm × 0.2 mm) fabricated by a photopolymerization 3D printer. The absorption band at approximately 980 cm^−1^ [25], assigned to the out-of-plane deformation vibration of vinyl groups, was selected to monitor the consumption of reactive C=C bonds during photopolymerization. The absorption band at 1720 cm^−1^ corresponding to the C=O stretching vibration was used as an internal reference because it remained unchanged during curing. The DC value was calculated from the integrated area ratio of the C=C and C=O bands before and after curing.

(1) $$DegreeofConversion(\%)=\left[1-\frac{{\left(A_{C=C}/A_{C=O}\right)}_{t}}{{\left(A_{C=C}/A_{C=O}\right)}_{0}}\right]\times 100$$

where A_C=O_ and A_C=C_ represent the integrated areas of the carbonyl (C=O) absorption band at 1720 cm^−1^ and the vinyl (C=C) absorption band at 980 cm^−1^, respectively. The ratios of (A_C=C_/A_C=O_)_0_ and (A_C=C_/A_C=O_)_t_ correspond to the relative concentrations of unreacted C=C bonds before and after curing, respectively.

The gel fraction and swelling ratio were measured to evaluate the crosslinking degree of the cured polymer networks. Post-cured specimens (2.0 mm × 2.0 mm × 25 mm) were dried in a vacuum oven at 60 °C for 24 h and weighed to obtain the initial dry weight (w_1_). The samples were then immersed in 30 mL of acetone and gently shaken at room temperature for 48 h. After equilibrium swelling was reached, the specimens were removed from the solvent, and excess solvent on the surface was carefully blotted with filter paper before recording the swollen weight (w_2_). Subsequently, the specimens were dried again in a vacuum oven at 50 °C for 24 h until a constant weight was achieved, and the final dry weight (w_3_) was recorded. Five parallel specimens were tested for each formulation.

The gel fraction was determined from Equation (2):

(2) $$Gelfraction(\%)=\frac{W_{3}}{W_{1}}\times 100$$

The swelling ratio was determined from Equation (3):

(3) $$Swellingratio(\%)=\frac{W_{2}-W_{1}}{W_{1}}\times 100$$

where w_1_, w_2_, and w_3_ represent the initial dry weight, the equilibrium swollen weight, and the dry weight after solvent extraction and redrying to a constant weight, respectively.

The density of the uncured liquid resin (ρ_1_) was measured using a glass hydrometer based on Archimedes’ principle. The density value was directly obtained from the calibrated scale after the hydrometer reached equilibrium in the resin. All density measurements were conducted at room temperature (25 °C).

The densities of the printed samples were determined using a digital analytical balance (FA1104J, Shunyuhenping Scientific Instrument Int., Shanghai, China) equipped with a density determination kit based on Archimedes’ principle. Specimens (2.0 mm × 2.0 mm × 25 mm) were first weighed in air (M1) and subsequently weighed while immersed in distilled water (M2). The distilled water temperature was recorded during measurement, and the corresponding water density (ρ_T_) was used for calculation. The density of the cured sample (ρ_2_) was calculated according to Equation (4):

(4) $$\rho_{2}=\left[\frac{M_{1}}{M_{1}-M_{2}}\right]\times \rho_{T}$$

where M_1_ is the mass of the cured sample in air, M_2_ is the mass of the cured sample in distilled water, and ρ_T_ is the density of distilled water at temperature T.

The shrinkage rate was calculated using Equation (5) [24].

(5) $$\mathrm{VS}\;(\%)=\left[\frac{\rho_{2}-\rho_{1}}{\rho_{2}}\right]\times 100$$

where ρ_2_ and ρ_1_ are the specific gravities of the printed solid and liquid (before printing), respectively.

The Shore hardness of each specimen was measured using a D-type Shore hardness tester (HLX-D, Shanghai Yanrun Light Machine Technology Co., Ltd., Shanghai, China) according to ASTM D2240 [26]. Three specimens with dimensions of 15 mm × 15 mm × 5 mm were printed for each formulation.

Impact properties were evaluated according to ISO 180:2023 [27] using an impact testing machine (PTM7000-B1, Shenzhen Suns Technology Stock Co., Ltd., Shenzhen, China). Seven notched impact specimens with dimensions of 80 mm × 10 mm × 4 mm and a notch depth of 2 mm were prepared for each printable system, and the average impact strength was reported.

Flexural properties were measured using a universal testing machine (AGS-10KN, Shimadzu, Kyoto, Japan) under a three-point bending configuration according to ASTM D790 [28]. Seven specimens (80 mm × 10 mm × 4 mm) were printed per formulation. The span length was 64 mm, and the crosshead speed was 5 mm/min. The flexural strength (FS) and flexural modulus (FM) were obtained from the instrument software.

Tensile properties were evaluated according to ISO 527-2 [29] using a universal testing machine (BT1-FR010TH A50, ZWICK, Ulm, Germany) equipped with a 10 kN load cell. The tensile specimens were printed in a dumbbell configuration with overall dimensions of 150 mm × 20 mm × 4 mm. The tensile test was conducted at a crosshead speed of 5 mm/min, and five specimens were tested for each formulation. The average value was reported.

Dynamic mechanical analysis (DMA) was conducted using a DMA242E instrument (NETZSCH, Selb, Germany) in the temperature-sweep mode. The samples were heated from 35 °C to 250 °C at a ramp rate of 3 K min^−1^, during which the storage modulus (E′) and loss factor (tan δ) were continuously recorded. The measurements were carried out at a fixed frequency of 1 Hz, with the applied strain maintained at a sufficiently low level to ensure that the response remained within the linear viscoelastic regime of the material. Given that the crosslinked network topology directly mirrors the architecture of the cured resin, the crosslinking density ϑ e of the hybrid resins was additionally determined. This parameter was estimated according to Equation (6) [30].

(6) $$\vartheta e=\frac{Eʹ}{3RT}$$

where Eʹ is the storage modulus in the rubbery state (T g + 50 °C, where T g is the glass transition temperature), R is the universal gas constant, and T is the absolute temperature at T g + 50 °C.) According to the DMA curves, the storage modulus (E′) of all samples reached a relatively stable region after the glass transition, indicating that Tg + 50 °C was suitable for comparative evaluation. Considering the limitations of rubber elasticity theory for heterogeneous crosslinked acrylate networks, the calculated crosslinking density values were used only to compare the relative changes among different formulations rather than as absolute values.

Thermal stability of the LCD-printed resin specimens was evaluated by thermogravimetric analysis (TGA) using a TG209 instrument (NETZSCH, Germany) under a nitrogen (N_2_) atmosphere. The printed specimens were first subjected to UV curing and thermal post-curing at 150 °C for 2 h, followed by drying in a vacuum oven at 80 °C for 24 h to remove residual moisture. Subsequently, the cured resin specimens were heated from 35 to 600 °C at a heating rate of 20 K/min under a nitrogen flow, and the corresponding weight loss behavior was recorded.

The heat deflection temperature (HDT) of the printed samples was determined according to ISO 75 [31] using a three-point bending configuration (VTM1300-A1, Shenzhen Suns Technology Stock Co., Ltd., Shenzhen, China). The specimens with dimensions of 80 mm × 10 mm × 4 mm were fabricated using an LCD-based photopolymerization 3D printer and subsequently post-cured under UV light prior to testing. The HDT was measured under constant bending stresses of 0.45 MPa and 1.80 MPa. During the test, the temperature was increased at a rate of 2 °C/min, and the temperature at which the specimen reached a deflection of 0.34 mm was recorded as the HDT value. For each formulation, at least three specimens were tested, and the average value was reported.

Statistical analysis was performed using IBM SPSS Statistics 27.0. All experimental data were presented as mean ± standard deviation (SD). One-way analysis of variance (one-way ANOVA) was conducted to evaluate the differences among groups, followed by Tukey’s multiple comparison test for pairwise comparisons. A significance level of α = 0.05 was used, and differences were considered statistically significant when *p* < 0.05.

## 3. Results

### 3.1. Optimization of THEICTA/DMAA Formulation

#### 3.1.1. Viscosity of the Resins

**Figure 1:**
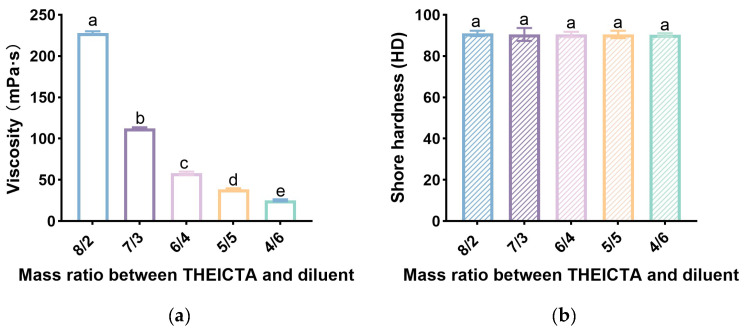
(**a**) Viscosities of the hybrid resins; (**b**) Shore hardness of the 3D-printed hybrid resins. Error bars represent standard deviation (SD) with *n* = 3. Statistical analysis was performed using Tukey’s multiple comparison test (*p* < 0.05). Different letters indicate statistically significant differences among groups.

As presented in Figure 1a, all resin formulations exhibited relatively low viscosity at 25 °C, which is favorable for LCD-based vat photopolymerization. With increasing DMAA content, the viscosity of the resin gradually decreased from approximately 225 mPa·s for the 8:2 formulation to around 25 mPa·s for the 4:6 formulation.

#### 3.1.2. The Degree of Conversion

**Figure 2:**
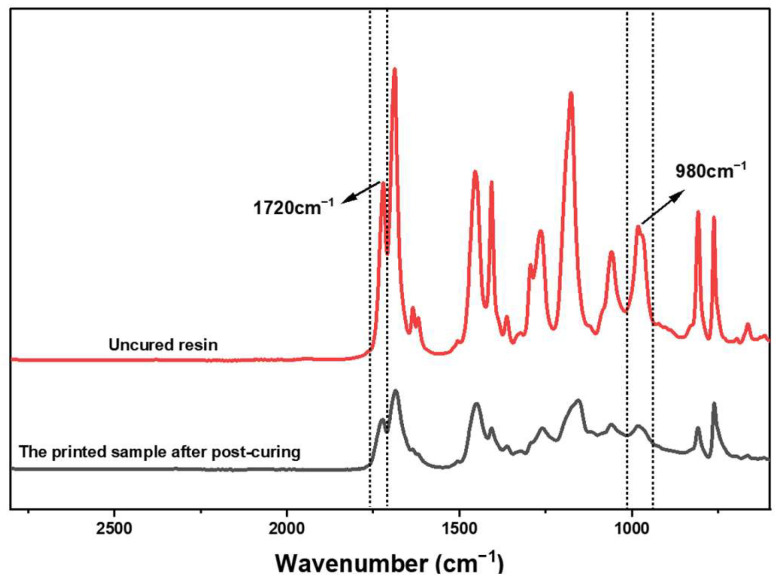
ATR-FTIR spectra of the uncured resin and the printed sample after post-curing.

Figure 2 shows the FTIR spectra of the uncured resin and the corresponding 3D-printed specimen after curing. The characteristic absorption band at 980 cm^−1^, attributed to the out-of-plane bending vibration of acrylate C=C bonds, decreases significantly after printing and post-curing, indicating extensive consumption of reactive double bonds during photopolymerization. As can be seen in Table 3, the results obtained from different resin formulations revealed that the degree of conversion (DC) exhibited a significant increasing trend with increasing DMAA content, gradually increasing from 37.4% to 87.7%.

#### 3.1.3. The Gel Fraction and Swelling Ratio

Table 3 presents the gel fraction and swelling ratio of the hybrid resins. For the THEICTA/DMAA system, the gel content of all samples remained above 96%. The swelling ratio gradually increased from 2.34% to 7.96%.

#### 3.1.4. Volumetric Shrinkage

The volumetric shrinkage of the resin systems with different ratios is summarized in Table 4. As shown in Table 4, the shrinkage increased progressively from 7.2 ± 0.6% to 15.4 ± 0.7% with increasing DMAA content.

#### 3.1.5. Shore Hardness

The Shore hardness of the printed specimens is shown in Figure 1b. Interestingly, despite the continuous decrease in resin viscosity with increasing DMAA content, all cured samples maintained similar hardness values of approximately 90 HD, and no obvious variation was observed among different formulations. These results indicate that DMAA incorporation effectively improves resin fluidity while maintaining the surface hardness of the printed materials.

#### 3.1.6. Impact Strength

The impact strength test was conducted on all printable samples under different THEICTA/DMAA mass ratios. As shown in Table 4, with increasing THEICTA content, the impact strength of the material exhibited an overall upward trend, increasing from 1.42 kJ/m^2^ at a 4:6 ratio to 2.50 kJ/m^2^ at an 8:2 ratio. These results indicated that increasing the proportion of THEICTA in the system effectively enhances the impact resistance of the resin system.

#### 3.1.7. Flexural Properties

**Figure 3:**
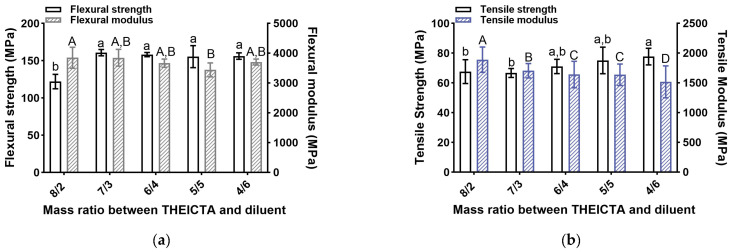
(**a**) Flexural properties (strength and modulus) of the 3D-printed hybrid resins; (**b**) tensile properties (strength and modulus) of the 3D-printed hybrid resins. Error bars represent standard deviation (SD) with *n* = 7. Statistical analysis was performed using Tukey’s multiple comparison test (*p* < 0.05). Different letters indicate statistically significant differences among groups.

As can be seen in Figure 3a, as the resin ratio changed from 8:2 to 7:3, the flexural strength increased significantly from 122.04 MPa to 160.80 MPa. With further DMAA increase, the flexural strength slightly decreased but remained relatively stable. In the contrast, the flexural modulus showed a slight decreasing trend overall, declining from 3850.96 MPa at 8:2 to 3443.95 MPa at 5:5.

#### 3.1.8. Tensile Properties

Figure 3b presents the tensile properties of resin samples formulated with different THEICTA/DMAA ratios, and the corresponding data are summarized in Table 5. As the DMAA content increased from 8:2 to 4:6, the tensile strength increased from 67.6 MPa to 77.7 MPa, accompanied by an increase in elongation at break from 4.1% to 5.5%. In contrast, the tensile modulus gradually decreased from 1890.0 MPa to 1518.3 MPa.

#### 3.1.9. Dynamic Mechanical Properties

**Figure 4:**
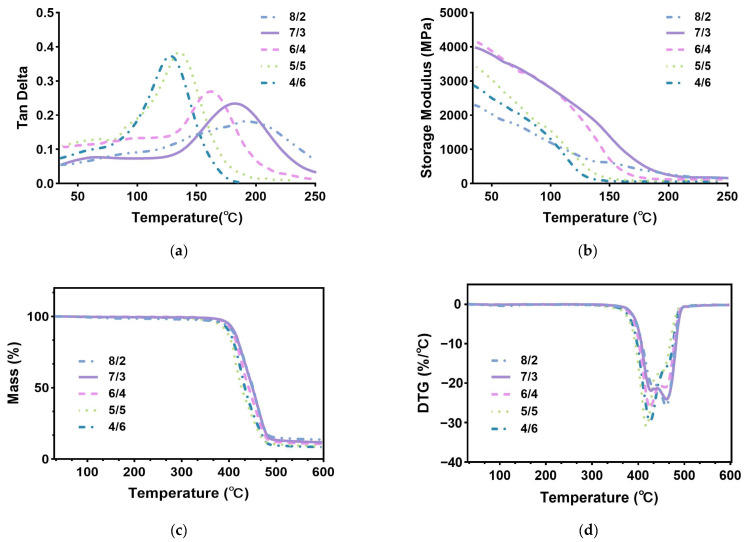
Dynamic mechanical analysis (DMA) curves of the 3D-printed hybrid resins: (**a**) loss factor (tan δ); (**b**) storage modulus (E′); (**c**) thermogravimetric (TG) curves; (**d**) derivative thermogravimetric (DTG) curves.

Figure 4a and Figure 4b present the tan δ and storage modulus of printable samples, respectively. The glass transition temperature (T g) was identified at the peak of the corresponding tan δ curve. As the content of THEICTA increased from 40 to 80 wt.%, T g showed a significant increase from 128.1 to 191.3 °C. The storage modulus demonstrates a pronounced formulation dependence across the entire temperature range, with the most significant differences observed in the glassy region. A higher THEICTA content endows the system with a higher E′ except the 8:2 formulation which showed the lowest modulus. In the rubbery plateau region, samples with high THEICTA content still maintain a relatively high E′. The crosslinking density, calculated based on rubber elasticity theory and shown in Table 6, revealed a trend of initially increasing and then decreasing with varying DMAA content, with the 7:3 system exhibiting the highest crosslinking density.

#### 3.1.10. Thermal Stability of the 3D-Printed Samples

The thermal stabilities of the various resin systems were investigated using TGA/DTG, and are summarized in Figure 4c, d. The weight losses, as well as various pyrolysis steps up to 600 °C in N_2_, are shown in Table 7. From the TGA curves, all samples exhibit a major decomposition process in the temperature range of approximately 350–450 °C. The overall thermal stability increases with higher THEICTA content. Meanwhile, the char yield at 600 °C gradually rises from 8.38% for 4:6 to 13.57% for 8:2, indicating that higher THEICTA content promotes the formation of a more stable carbonaceous structure at elevated temperatures. The DTG curves show that the maximum decomposition rate peaks for all samples are mainly located between 420 and 470 °C. These peaks shift slightly to higher temperatures with THEICTA increase.

#### 3.1.11. Heat Distortion Temperature

**Figure 5:**
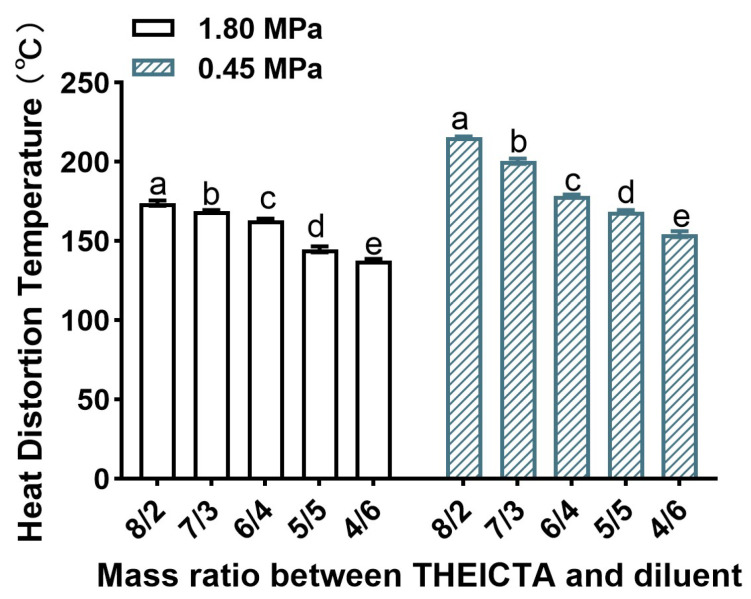
Heat deflection temperature of the 3D-printed hybrid resins. Error bars represent standard deviation (SD) with *n* = 3. Statistical analysis was performed using Tukey’s multiple comparison test (*p* < 0.05). Different letters indicate statistically significant differences among groups.

Figure 5 shows the heat distortion temperatures (HDTs) of resin systems with varying THEICTA/DMAA mass ratios under applied loads of 0.45 MPa and 1.80 MPa. It can be observed that, as the mass ratio of THEICTA to DMAA decreases from 8:2 to 4:6, the HDT under 0.45 MPa decreases from approximately 215 °C to 155 °C, while under 1.80 MPa, the HDT decreases from approximately 173 °C to 138 °C. Furthermore, for all compositions, the HDT measured at 0.45 MPa is consistently higher than that at 1.80 MPa. Although the trends of both curves are similar, the reduction in HDT is more pronounced at the higher applied load.

Among the investigated formulations, the 6:4 composition exhibited a favorable balance between viscosity, volumetric shrinkage, mechanical properties, and thermal stability, making it a suitable candidate for further modification.

### 3.2. Effect of Partial DMAA Substitution with DCPDA

#### 3.2.1. Viscosity of the Resins

**Figure 6:**
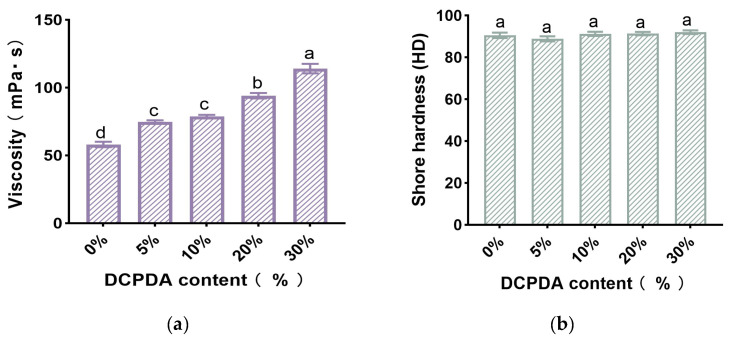
(**a**) Viscosities of the hybrid resins with different concentrations of DCPDA; (**b**) Shore hardness of the 3D-printed hybrid resins with different concentrations of DCPDA. Error bars represent standard deviation (SD) with *n* = 3. Statistical analysis was performed using Tukey’s multiple comparison test (*p* < 0.05). Different letters indicate statistically significant differences among groups.

Based on the viscosity measurements of resin systems under different DCPDA substitution ratios shown in Figure 6a, the viscosity exhibited a progressive increase with increasing DCPDA content. When the mass ratio between THEICTA and DMAA was 6:4, the viscosity was 58 mPa·s. As the substitution ratio of DCPDA increased to 30%, the viscosity rose to 114 mPa·s.

#### 3.2.2. The Degree of Conversion

As shown in Table 8, the double-bond conversion of all formulations remained in the range of 57.6–61.1%, and no statistically significant differences were observed among the samples (*p* > 0.05).

#### 3.2.3. The Gel Fraction and Swelling Ratio

Table 8 shows for the DCPDA-modified system, all samples exhibited gel contents above 97.8%. As the DCPDA content increased from 0 wt.% to 20 wt.%, the swelling ratio decreased from 3.3% to 1.8%. However, with a further increase in DCPDA content to 30 wt.%, the swelling ratio increased again to 3.1%.

#### 3.2.4. Volumetric Shrinkage

Table 9 illustrates the results of polymerization shrinkage. Compared with the 6:4 resin system the polymerization shrinkage of the resin decreases significantly as the DCPDA content increases. Notably, the polymerization shrinkage of the resin bottomed out at 9.7% when the DCPDA content was set to 10 wt.% Further increases in DCPDA content beyond this point essentially reached a stable state.

#### 3.2.5. Shore Hardness

The results are presented in Figure 6b. With increasing replacement of DMAA by DCPDA, the Shore hardness of the cured materials remained relatively constant within the range of 88–92 HD. Although slight fluctuations were observed, no statistically significant differences were detected among the formulations (*p* > 0.05).

#### 3.2.6. Impact Strength

The impact strength test was conducted on all printable samples under the different substitution ratios of DCPDA for DMAA. As shown in Table 6, with the introduction of DCPDA, the impact strength of the resin system initially decreased slightly, reached a maximum at a substitution level of 10%, and then gradually declined with further increase in DCPDA content.

#### 3.2.7. Flexural Properties

**Figure 7:**
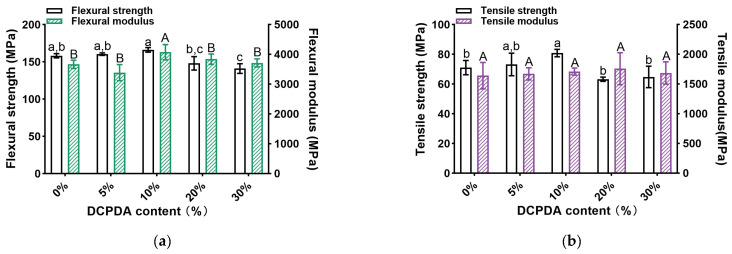
(**a**) Flexural properties (strength and modulus) of the 3D-printed hybrid resins with different concentrations of DCPDA; (**b**) tensile properties (strength and modulus) of the 3D-printed hybrid resins with different concentrations of DCPDA. Error bars represent standard deviation (SD) with *n* = 7. Statistical analysis was performed using Tukey’s multiple comparison test (*p* < 0.05). Different letters indicate statistically significant differences among groups.

As can be seen in Figure 7a, With increasing DCPDA content, the flexural strength and modulus of the material initially increased and then decreased. The highest flexural strength (166.14 ± 3.07 MPa) and modulus (4076.51 ± 256.38 MPa) were observed at a DCPDA content of 10%.

#### 3.2.8. Tensile Properties

Figure 7b demonstrates the tensile properties of printed resin samples formulated with different concentrations of DCPDA. The tensile properties of these samples are summarized in Table 10. With increasing DCPDA content, the tensile strength, tensile modulus, and elongation at break initially increased and then decreased. The highest tensile strength and elongation at break were obtained at 10 wt.%. DCPDA, while the maximum tensile modulus was achieved at 20 wt.% DCPDA.

#### 3.2.9. Dynamic Mechanical Properties

**Figure 8:**
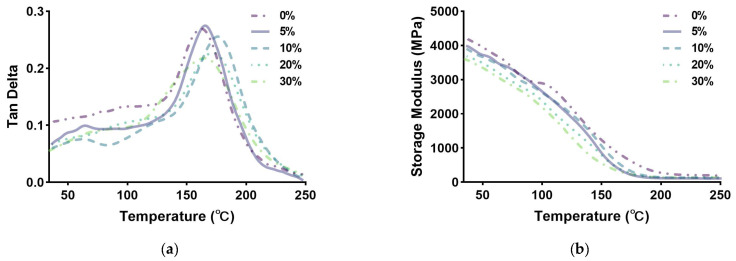

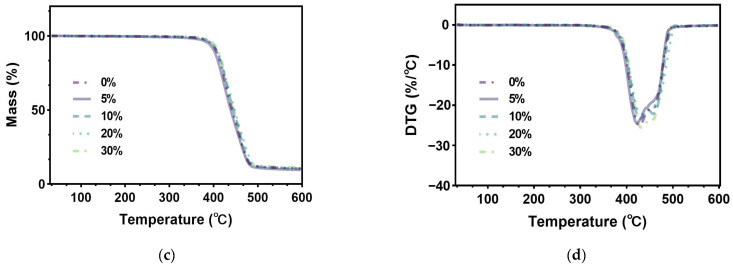
Dynamic mechanical analysis (DMA) curves of the 3D-printed hybrid resins with different concentrations of DCPDA: (**a**) loss factor (tan δ); (**b**) storage modulus (E′). (**c**) Thermogravimetric (TG) curves of the 3D-printed hybrid resins with different concentrations of DCPDA; (**d**) derivative thermogravimetric (DTG) curves of the 3D-printed hybrid resins with different concentrations of DCPDA.

Figure 8a and Figure 8b present the tan δ and storage modulus of printable samples with different concentrations of DCPDA, respectively. DMA showed that the glass transition temperature (T g) of the 6:4 system increased with DCPDA incorporation, reaching a maximum of 176.2 °C at 10% substitution in Table 11, and then decreased at higher contents. The storage modulus decreased after the incorporation of DCPDA. The crosslinking density, calculated based on rubber elasticity theory and shown in Table 11, ranging from 8.93 × 10^3^ mol·m^−3^ to 1.10 × 10^4^ mol·m^−3^. The highest values are observed at 20% and 30% DCPDA.

#### 3.2.10. Thermal Stability of the 3D-Printed Samples

The thermal stabilities of the 3D-printed hybrid resins with different concentrations of DCPDA are investigated using TGA/DTG and the results are shown in Figure 8a,b. Table 12 presents the TGA results of 3D-printed hybrid resins with different concentrations of DCPDA. All samples exhibited similar degradation behavior, indicating that the incorporation of DCPDA does not significantly change the degradation mechanism. The onset degradation temperature ranges from 388.3 °C to 399.3 °C, suggesting good thermal stability for all samples. Compared with a pure 6:4 system, the incorporation of 5% DCPDA led to a slight decrease in thermal stability. As the DCPDA content increased, T_max_ shifted slightly to higher temperatures, reaching 433.6 °C. When the DCPDA content was 30%, the char yield at 600 °C reached 11.19%.

#### 3.2.11. Heat Distortion Temperature

**Figure 9:**
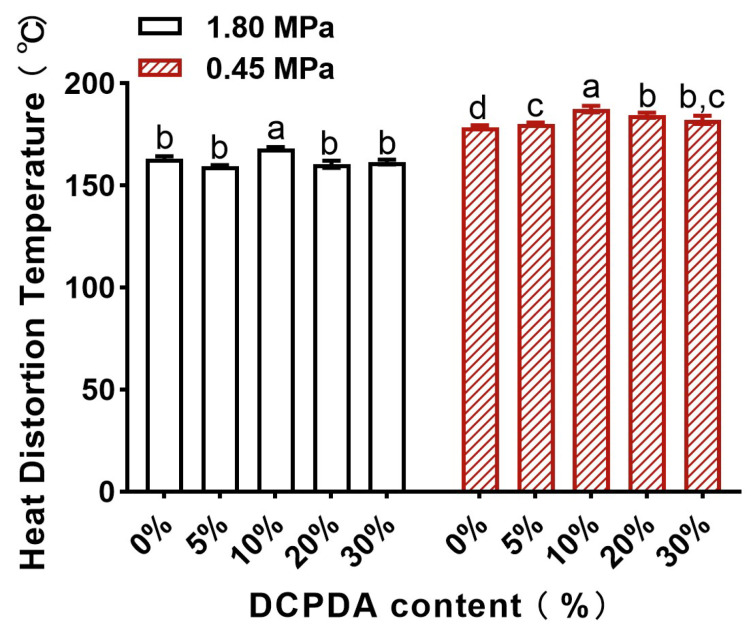
Heat deflection temperature of the 3D-printed hybrid resins with different concentrations of DCPDA. Error bars represent standard deviation (SD) with *n* = 3. Statistical analysis was performed using Tukey’s multiple comparison test (*p* < 0.05). Different letters indicate statistically significant differences among groups.

Figure 9 shows the HDT of resin systems with varying substitution ratios of DCPDA for DMAA under applied loads of 0.45 MPa and 1.80 MPa. With the addition of DCPDA, it can be observed that the heat deflection temperature (HDT) of the material initially increased and then decreased, reaching a maximum at a DCPDA content of 10%.

#### 3.2.12. Printability and Reusability Evaluation

**Figure 10:**
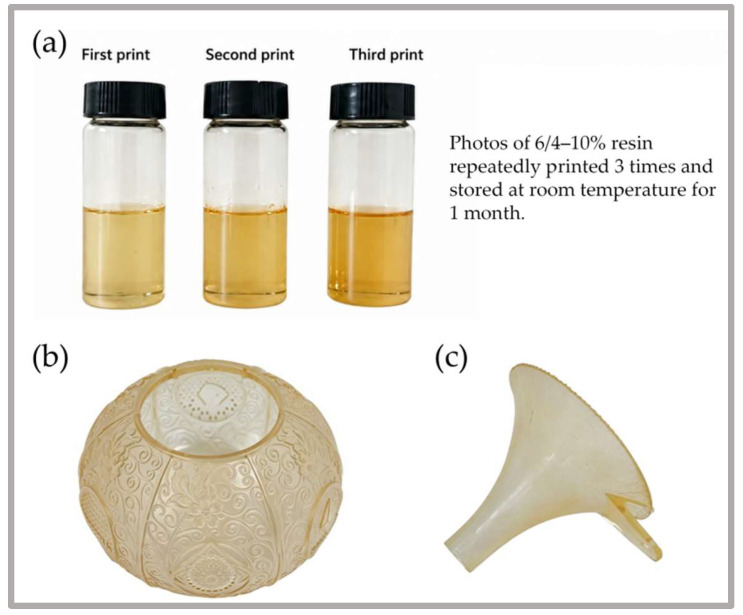
Storage stability and 3D printing specimens of the 6/4–10% resin. (**a**) Digital photographs of the 6/4–10% resin after three repeated printing cycles and one month of room-temperature storage (from left to right: first print, second print, third print). (**b**) 3D-printed spherical decorative artifact with intricate patterns fabricated using the as-prepared resin. (**c**) Complex horn-shaped structural part manufactured by stereolithography with the target resin.

The practical printability and reusability of the 6/4–10% resin was evaluated by fabricating complex structures, including an embossed hollow lampshade and a thin-walled curved horn, using LCD 3D printing. As shown in Figure 10, the printed objects exhibited excellent surface quality, high structural fidelity, and accurate reproduction of intricate features. After three consecutive printing cycles and one month of storage at room temperature, the recycled resins remained homogeneous without observable phase separation, sedimentation, or gelation, showing only slight yellowing. These results demonstrate the excellent printability, reusability, and storage stability of the developed resin system.

## 4. Discussion

Viscosity plays a critical role in the printing quality, resolution, and thickness accuracy of 3D-printed objects [32]. Low-viscosity formulations provide optimal processing performance for fabricating objects using 3D printers. Low-viscosity oligomers can reduce the viscosity of the resin, thereby altering its rheological properties [33]. The viscosity of the resin was first investigated by varying the DMAA content. DMAA is a low-viscosity monofunctional acrylamide monomer that is commonly used as a reactive diluent. Therefore, increasing the DMAA content gradually reduced the viscosity of the resin system. Based on the 6:4 formulation, DMAA was partially replaced by DCPDA. Owing to its rigid bicyclic structure and higher intrinsic viscosity, the incorporation of DCPDA led to a gradual increase in viscosity.

For the THEICTA/DMAA resin system, the degree of conversion (DC) increased significantly from 37.4% to 87.7% as the DMAA content increased from 20 wt.% to 60 wt.%. The reduced viscosity improved the mobility of reactive species during photopolymerization, thereby facilitating the curing reaction. In contrast, the introduction of DCPDA had little influence on the DC, indicating that DCPDA mainly affected the network structure rather than the overall polymerization conversion.

To further evaluate the integrity and compactness of the cured network structure, the gel content and swelling ratio of the samples were investigated. For the THEICTA/DMAA system, all samples exhibited gel contents higher than 96%, indicating the formation of stable three-dimensional crosslinked networks after photopolymerization. With increasing DMAA content, the gel content showed a slight decrease, while the swelling ratio increased significantly from 2.4% to 8.0%. This result suggests that the incorporation of monofunctional DMAA reduces the effective crosslinking density within the polymer network, leading to a relatively looser network structure. For the DCPDA-modified system, increasing the DCPDA content had a negligible effect on the gel content, whereas the swelling ratio decreased from 3.3% to 1.8%. This behavior can be attributed to the bifunctional acrylate groups of DCPDA, which increase the concentration of crosslinking points within the network. Moreover, the rigid dicyclodecane structure restricts polymer chain mobility and reduces the free volume, resulting in the formation of a more compact crosslinked network. However, when the DCPDA content was further increased to 30 wt.%, the swelling ratio increased again to 3.1%, accompanied by a slight decrease in gel content. This phenomenon may be related to the steric hindrance caused by excessive rigid structures and the reduced network homogeneity, which could generate additional local free volume and facilitate solvent penetration.

Volumetric shrinkage is an important parameter affecting the dimensional accuracy of photocured materials. Excessive shrinkage can cause warpage and reduce printing precision [34]. The volumetric shrinkage of photocurable resins is closely related to the concentration of polymerizable double bonds [35]. In the present system, DMAA possesses a higher double-bond concentration (1.008 mol of C=C bonds per 100 g resin) than THEICTA (0.651 mol of C=C bonds per 100 g resin). Therefore, the volumetric shrinkage increased gradually with increasing DMAA content. The relatively large variation in volumetric shrinkage for the 7/3 formulation may be associated with the substantial increase in double-bond conversion at this stage, which leads to increased scatter in shrinkage behavior. For the DCPDA-modified formulations, the incorporation of DCPDA reduced the volumetric shrinkage. This result can be attributed to the lower double-bond concentration of DCPDA (0.786 mol of C=C bonds per 100 g resin) compared with DMAA, together with the rigid bicyclic structure of DCPDA, which limits structural contraction during curing.

Hardness is related to both the rigidity of polymer chains and the structure of the cured network [36]. Although the 8:2 formulation exhibited slightly higher hardness values, the differences among all formulations were not statistically significant. Similarly, the introduction of DCPDA did not produce a significant change in hardness. These results indicate that variations in resin composition had only a limited influence on the surface hardness of the cured materials.

The impact strength reflects the ability of a material to resist sudden fracture under impact loading and is closely related to the network structure of the cured polymer [7]. As the THEICTA content increased, the impact strength gradually increased. This behavior can be attributed to the higher proportion of trifunctional acrylate groups in the system, which promotes the formation of a more highly crosslinked network. In addition, the rigid isocyanurate structure of THEICTA contributes to the mechanical stability of the cured material. With increasing DMAA content, the proportion of monofunctional monomer increased, leading to a reduction in effective crosslink density and consequently lower impact strength. For the DCPDA-modified formulations, the impact strength initially increased and reached a maximum at 10 wt.% DCPDA. The improvement may be associated with the formation of a more compact network structure, as supported by the lower swelling ratio. However, further increasing the DCPDA content resulted in a gradual decrease in impact strength. Excessive incorporation of the rigid bicyclic structure increased the brittleness of the network, thereby reducing the resistance to impact fracture.

When the DMAA content increases from approximately 20% to 30%, the flexural strength of the material shows a pronounced improvement. This enhancement can be mainly attributed to DMAA, as a low-molecular-weight acrylamide monomer, significantly reducing the initial viscosity of the resin system, thereby improving the diffusion of monomers and free radicals during the polymerization process [37]. The reduced viscosity facilitates a higher degree of double-bond conversion and promotes a more complete polymerization reaction, leading to a more complete curing process and improved mechanical performance. However, as the DMAA content continues to increase, both the flexural strength and flexural modulus exhibit a gradual decline. This behavior is primarily associated with the mono-functional nature of DMAA. An excessive proportion of DMAA reduces the number of effective cross-linking sites within the system, thereby decreasing the overall cross-link density of the polymer network [38]. A lower cross-link density weakens the constraints between polymer chains and facilitates segmental mobility, which consequently reduces the stiffness and load-bearing capability of the material. When a small amount of DCPDA is introduced into the system, the rigidity of the polymer chains is enhanced and the cross-linking point density increases. Meanwhile, the incorporation of DCPDA reduces volumetric shrinkage and increases network rigidity, which contributes to the improvement of flexural properties and consequently improving the flexural strength and flexural modulus of the material. However, further increasing the DCPDA content does not lead to a significant increase in crosslinking density but instead results in a noticeable decline in flexural performance. This phenomenon can be attributed to the excessive introduction of rigid backbone structures, which restrict chain segment mobility and induce material embrittlement, ultimately deteriorating the flexural properties.

**Figure 11:**
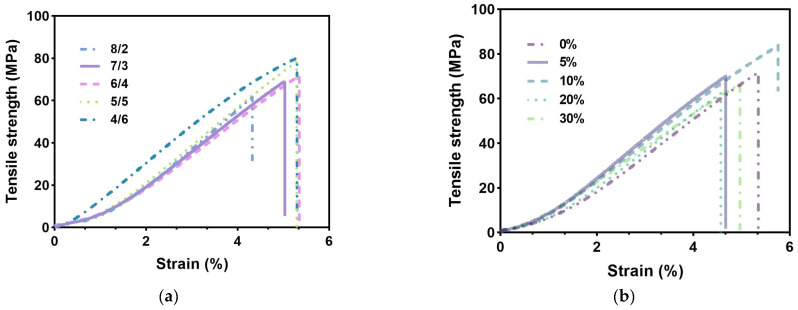
Tensile stress–strain curves of photocurable resin systems. (**a**) Resins with different THEICTA/DMAA mass ratios. (**b**) Resins with varying DCPDA mass fractions.

Tensile properties reflect the mechanical response of materials under tensile loading and provide important information regarding their load-bearing capability and deformation behavior. For the THEICTA/DMAA system, the tensile strength increased continuously with increasing DMAA content, despite the reduction in crosslink density. This behavior is mainly attributed to the significant increase in double-bond conversion. As the DMAA content increased, the viscosity decreased and the DC increased from 37.4% to 87.7%, indicating a more complete curing process. The higher conversion contributed to the improvement in tensile strength and compensated for the reduction in crosslink density caused by the monofunctional monomer. Meanwhile, increasing DMAA content introduced more flexible molecular units into the network, resulting in a gradual decrease in tensile modulus and a continuous increase in elongation at break [39]. As shown in Figure 11a, the stress–strain curves shifted toward higher strain values with increasing DMAA content, indicating enhanced deformability of the cured materials. For the 6/4 THEICTA/DMAA formulation, further improvement in tensile performance was achieved by incorporating DCPDA. The bifunctional structure of DCPDA increased the density of effective crosslinking points, while its rigid bicyclic structure enhanced the rigidity of the polymer network. In addition, the reduced swelling ratio suggested the formation of a more compact crosslinked structure. As a result, the formulation containing 10 wt.% DCPDA exhibited the highest tensile strength and tensile modulus. However, when the DCPDA content exceeded 10 wt.%, both tensile strength and elongation at break decreased. As shown in Figure 11b, the 20 wt.% and 30 wt.% DCPDA formulations exhibited shorter plastic deformation regions and lower elongation at break, indicating increased brittleness.

Dynamic mechanical analysis (DMA) is a suitable and effective technique to study the viscoelastic properties of UV-cured polymeric materials. The DMA data allow observations of changes in storage modulus, glass transition temperature (T g), and cross-link density. The tan δ peak reflects the energy dissipation behavior of the material during the glass transition and can also provide insight into the uniformity of the polymer network structure [40]. With increasing THEICTA content, the tan δ peak shifts toward higher temperatures, corresponding to an increase in the glass transition temperature (T g). This is mainly attributed to the high functionality and rigid structure of THEICTA, which promotes the formation of a denser cross-linked network during curing, thereby increasing the cross-linking density and restricting segmental motion of polymer chains [41]. Meanwhile, an increase in cross-linking density is generally accompanied by a decrease in the tan δ peak height, explaining the lower tan δ values observed in systems with higher THEICTA content. For the 8:2 system, the relatively rapid curing rate may induce localized premature gelation during network formation. This may contribute to structural heterogeneity in the network, leading to a noticeable broadening of the tan δ peak [40,42]. Partial replacement of DMAA with DCPDA results in the content of 10% DCPDA formulation exhibiting the highest T g. At low concentrations, the rigid cyclic structure of DCPDA effectively restricts polymer chain segment motion, thus increasing T g. However, when the DCPDA content is further increased, greater steric hindrance is introduced. This may increase the free volume and reduce the uniformity of the cross-linked network, facilitating segmental motion and, consequently, decreasing T g. In the 3D-printed hybrid resin systems, the 8:2 system exhibits the lowest storage modulus in the glassy region, which is mainly attributed to the relatively high content of THEICTA. The steric hindrance associated with the bulky molecular structure of THEICTA restricts the effective contact of reactive groups, which may contribute to a less homogeneous cross-linked network [42]. Consequently, although its cross-linking density is not the lowest, the presence of network inhomogeneity leads to a reduced storage modulus. Further increases in DMAA content lead to a decrease in the storage modulus, which can be attributed to a reduction in crosslink density [43]. In the 6:4 resin system, partially replacing DMAA with DCPDA results in the decrease in the storage modulus in the glassy region. This behavior may be associated with the rigid nature of the DCPDA monomer, whose bulky cyclic structure introduces pronounced steric hindrance during polymerization, resulting in a less homogeneous cross-linked network and consequently a lower storage modulus [44].

The higher the concentration of THEICTA, the higher the temperature of thermal decomposition, which is attributed to isocyanurate rings from THEICTA. The resin system of 7:3 exhibits a higher onset decomposition temperature, which can be attributed to its highest crosslink density among the studied systems. The dense network effectively restricts polymer chain mobility and enhances the overall rigidity of the network structure, thereby increasing resistance to thermal scission and shifting the decomposition process toward higher temperatures. Consequently, the resin system of 8:2 shows a slightly lower onset decomposition temperature than 7:3. The 8:2 system exhibits a higher char yield than 7:3, which is attributed to the presence of rigid isocyanurate heterocycles in the THEICTA units. These structures, which possess high thermal stability at elevated temperatures, can promote the formation of a stable carbonaceous structure [45], promoting the formation of a stable carbonaceous structure. The incorporation of 5% DCPDA slightly reduces the thermal stability of the system, which may be attributed to a minor decrease in crosslink density. As the DCPDA content increases, the bulky polycyclic structures introduced restrict the mobility of polymer chain segments, enhancing the rigidity of the molecular network and consequently improving the thermal stability of the material to some extent [46].

The heat distortion temperature of polymer materials is mainly determined by the crosslink density of the network structure [47] and the intrinsic rigidity of polymer chain segments [48]. In the present system, THEICTA, as a trifunctional monomer, can form a highly crosslinked three-dimensional network during photopolymerization. Its molecular structure contains highly rigid isocyanurate rings, which effectively limit the mobility of polymer chains [22]. At higher THEICTA contents, the system exhibits elevated HDT. This indicates that an increased proportion of rigid structural units enhances the thermal stability of the polymer network and imposes stronger constraints on segmental motion. As a result, higher temperatures are required to reach the specified deflection. Conversely, with increasing DMAA content, more relatively flexible molecular units are introduced into the resin network, which enhances chain mobility. Under combined thermal and mechanical loading, the polymer chains can undergo cooperative motion and local structural rearrangement more readily, leading to noticeable deformation at lower temperatures, which manifests as a reduction in HDT. At low concentrations, the incorporation of DCPDA enhances the rigidity of the polymer network due to the presence of the bulky tricyclodecane structure. Bulky cyclic substituents can significantly affect the packing efficiency of polymer chains [49]. However, upon further increase in DCPDA content, the enhanced steric hindrance may increase the free volume and compromise network homogeneity, rendering chain movement more facile. Furthermore, the differences in HDT under varying load conditions primarily arise from stress-dependent thermal deformation behavior. Under higher applied load (1.80 MPa), the material experiences greater internal bending stress, so a lower thermal activation energy is sufficient to induce viscoelastic deformation of polymer chains. Consequently, the sample reaches the specified deflection at a lower temperature, resulting in HDTs lower than those measured under 0.45 MPa.

**Figure 12:**
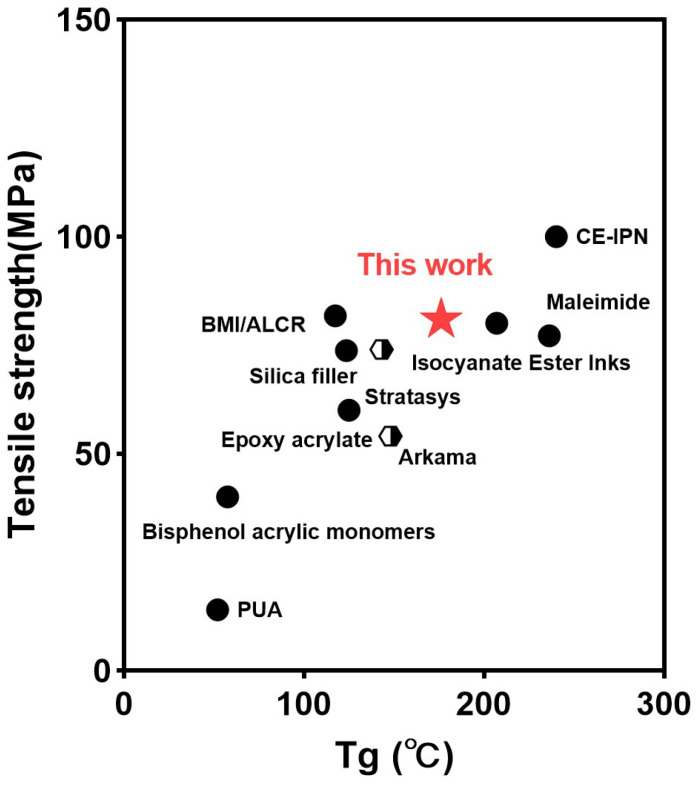
Comparison of mechanical and thermal properties of 3D-printed samples based on different resin. The black circles denote reported hybrid polymers, hexagonal symbols denote commercial 3D-printing resins, and the red star represents this work. (The complete literature data are summarized in Tables S1 and S2 of the Supporting Information).

To further evaluate the performance of the developed resin system, its mechanical and thermal properties were compared with representative photocurable resins reported in the literature and several commercial materials, as shown in Figure 12. Many reported high-temperature photocurable resins achieve elevated T g values at the expense of mechanical performance, whereas some high-strength systems exhibit relatively limited thermal resistance.

The optimized THEICTA/DMAA (6:4) formulation, in which 10 wt.% of DMAA was replaced by DCPDA, achieved a tensile strength of 80.9 MPa and a Tg of 176.2 °C, demonstrating a favorable balance between mechanical strength and thermal stability. As shown in the performance map, the developed resin system is positioned in the region combining relatively high tensile strength and high T g, outperforming many reported epoxy acrylate, polyurethane acrylate (PUA), and filler-reinforced photocurable systems.

Although some cyanate ester (CE) and bismaleimide (BMI)-based photocurable systems exhibit even higher T g values or mechanical strength, these materials generally require more demanding post-curing conditions and often suffer from limited processability. In comparison, the resin developed in this work provides competitive mechanical and thermal performance while maintaining good printability and relatively simple post-processing requirements, indicating its potential for high-performance additive manufacturing applications.

## 5. Conclusions

In this study, a high-performance photosensitive resin system suitable for LCD-based 3D printing was developed. By using THEICTA and DMAA as the primary components and optimizing the formulation, we obtained a low-viscosity resin that can be directly printed at room temperature. Partial substitution of DMAA with DCPDA increased the rigid structural content, leading to improved mechanical properties and thermal stability. The resulting resin exhibited a low viscosity of 78.7 mPa·s meeting the requirements for LCD photopolymerization 3D printing. When the DCPDA substitution ratio was 10%, the material exhibited a tensile strength of 80.9 MPa, a flexural strength of 166.14 MPa, and a T g of 176.2 °C. The heat deflection temperatures reached 168.1 °C under 1.80 MPa and 187.3 °C under 0.45 MPa. In addition, the printed parts showed low shrinkage (<10%) and good dimensional stability.

Overall, the proposed photosensitive resin effectively overcomes the limitations of conventional acrylate-based resins in LCD 3D printing and provides a promising strategy for developing high-strength and heat-resistant photocurable materials for advanced additive manufacturing applications.

## Figures and Tables

**Table 1:** Basic formulations of the hybrid resins (wt.%).

Resin Systems		Components (wt.%)	
	THEICTA	DMAA	TPO-L
8/2	78.4	19.6	2
7/3	68.6	29.4	2
6/4	58.8	39.2	2
5/5	49.0	49.0	2
4/6	39.2	58.8	2

**Table 2:** The hybrid resins with different concentrations of DCPDA (wt.%).

THEICTA	DMAA	DCPDA	TPO-L
58.8	39.2	0	2
58.8	37.2	2.0	2
58.8	35.3	3.9	2
58.8	31.4	7.8	2
58.8	27.4	11.8	2

**Table 3:** Volumetric shrinkage and the impact strength of the 3D-printed hybrid resins. Error bars represent standard deviation (SD). The sample sizes were *n* = 5 for DC and gel fraction and swelling ratio. Statistical analysis was performed using Tukey’s multiple comparison test with *p* < 0.05. Different letters indicate statistically significant differences among groups.

Resin Systems	DC (%)	Gel Fraction (%)	Swelling Ratio (%)
8/2	37.4 ± 1.3 ^d^	97.58 ± 1.11 ^a^	2.34 ± 0.66 ^c^
7/3	46.1 ± 2.6 ^c^	97.03 ± 0.90 ^a,b^	3.26 ± 1.00 ^b,c^
6/4	60.5 ± 3.7 ^b^	97.98 ± 1.39 ^a^	3.34 ± 0.99 ^b,c^
5/5	66.9 ± 2.8 ^b^	97.16 ± 0.30 ^a,b^	4.45 ± 0.40 ^b^
4/6	87.7 ± 1.2 ^a^	95.99 ± 0.85 ^b^	7.96 ± 0.69 ^a^

**Table 4:** Volumetric shrinkage and the impact strength of the 3D-printed hybrid resins. Error bars represent standard deviation (SD). The sample sizes were *n* = 5 for volumetric shrinkage and *n* = 7 for impact strength. Statistical analysis was performed using Tukey’s multiple comparison test with *p* < 0.05. Different letters indicate statistically significant differences among groups.

Resin Systems	Volumetric Shrinkage (%)	Impact Strength (KJ/m^2^)
8/2	7.2 ± 0.6 ^c^	2.50 ± 0.06 ^a^
7/3	11.5 ± 1.3 ^b^	2.01 ± 0.17 ^b^
6/4	12.3 ± 0.2 ^b^	1.69 ± 0.09 ^b,c^
5/5	15.0 ± 0.5 ^a^	1.56 ± 0.03 ^c,d^
4/6	15.4 ± 0.7 ^a^	1.42 ± 0.08 ^d^

**Table 5:** Tensile properties of 3D-printed hybrid resins. Error bars represent standard deviation (SD) with *n* = 5. Statistical analysis was performed using Tukey’s multiple comparison test (*p* < 0.05). Different letters indicate statistically significant differences among groups.

Resin Systems	Elongation at Break (%)	Tensile Strength (MPa)	Tensile Modulus (MPa)
8/2	4.1 ± 0.6 ^b^	67.6 ± 8.5 ^b^	1890.0 ± 212.1 ^A^
7/3	4.2 ± 0.4 ^b^	66.7 ± 3.5 ^b^	1705.0 ± 120.2 ^B^
6/4	4.7 ± 0.6 ^b^	71.1 ± 4.8 ^a,b^	1642.5 ± 222.0 ^C^
5/5	5.0 ± 0.7 ^a,b^	75.1 ± 8.9 ^a,b^	1637.5 ± 180.1 ^C^
4/6	5.5 ± 1.0 ^a^	77.7 ± 5.6 ^a^	1518.3 ± 168.3 ^D^

**Table 6:** Dynamic mechanical properties of the 3D-printed hybrid resins obtained from DMA.

Resin Systems	T g (°C)	E′ at T g + 50°C (MPa)	ϑ e (mol·m^−3^)
8/2	191.3	167.41	1.30 × 10^4^
7/3	182.2	180.61	1.43 × 10^4^
6/4	162.2	122.48	1.01 × 10^4^
5/5	135.4	85.63	7.49 × 10^3^
4/6	128.1	60.45	5.37 × 10^3^

**Table 7:** Thermal stability parameters of the 3D-printed hybrid resins obtained from TGA.

Resin Systems	Onset Degradation Temperature (°C)	T_10%_ (°C)	T _max_ (°C)	Char at 600 °C (%)
8/2	389.2	408.2	454.5	13.57
7/3	398.9	410.3	461.0	11.72
6/4	394.7	405.8	429.6	10.56
5/5	376.6	394.4	420.8	9.05
4/6	358.4	399.2	429.2	8.38

**Table 8:** Volumetric shrinkage and the impact strength of the 3D-printed hybrid resins with different concentrations of DCPDA. Error bars represent standard deviation (SD). The sample sizes were *n* = 5 for DC and gel fraction and swelling ratio. Statistical analysis was performed using Tukey’s multiple comparison test with *p* < 0.05. Different letters indicate statistically significant differences among groups.

Content of DCPDA	DC (%)	Gel Fraction (%)	Swelling Ratio (%)
0%	60.5 ± 3.7 ^a^	97.98 ± 1.39 ^a^	3.34 ± 0.99 ^a^
5%	60.0 ± 2.5 ^a^	98.00 ± 1.34 ^a^	2.93 ± 0.79 ^a,b^
10%	61.1 ± 3.1 ^a^	98.26 ± 0.31 ^a^	2.36 ± 0.35 ^a,b^
20%	57.6 ± 3.1 ^a^	98.84 ± 0.48 ^a^	1.83 ± 0.72 ^b^
30%	58.3 ± 2.9 ^a^	97.80 ± 0.69 ^a^	3.17 ± 0.66 ^a,b^

**Table 9:** Volumetric shrinkage and the impact strength of the 3D-printed hybrid resins with different concentrations of DCPDA. Error bars represent standard deviation (SD). The sample sizes were *n* = 5 for volumetric shrinkage and *n* = 7 for impact strength. Statistical analysis was performed using Tukey’s multiple comparison test with *p* < 0.05. Different letters indicate statistically significant differences among groups.

Content of DCPDA	Volumetric Shrinkage (%)	Impact Strength (KJ/m^2^)
0%	12.3 ± 0.2 ^a^	1.69 ± 0.09 ^a,b^
5%	11.5 ± 0.6 ^b^	1.59 ± 0.23 ^b^
10%	9.7 ± 0.1 ^c^	1.81 ± 0.08 ^a^
20%	9.8 ± 0.2 ^c^	1.10 ± 0.14 ^c^
30%	9.9 ± 0.4 ^c^	0.97 ± 0.19 ^d^

**Table 10:** Tensile properties of 3D-printed hybrid resins with different concentrations of DCPDA. Error bars represent standard deviation (SD) with *n* = 5. Statistical analysis was performed using Tukey’s multiple comparison test (*p* < 0.05). Different letters indicate statistically significant differences among groups.

Content of DCPDA	Elongation at Break (%)	Tensile Strength (MPa)	Tensile Modulus (MPa)
0%	4.7 ± 0.6 ^a,b^	71.1 ± 4.8 ^b^	1642.5 ± 222.0 ^A^
5%	4.7 ± 0.7 ^a,b^	73.2 ± 7.6 ^a,b^	1672.5 ± 102.1 ^A^
10%	5 ± 0.2 ^a^	80.9 ± 2.5 ^a^	1710.0 ± 59.4 ^A^
20%	3.9 ± 0.4 ^b^	63.3 ± 1.4 ^b^	1760.0 ± 268.7 ^A^
30%	4.2 ± 0.3 ^b^	64.8 ± 7.2 ^b^	1686.7 ± 187.2 ^A^

**Table 11:** Dynamic mechanical properties of the 3D-printed hybrid resins with different concentrations of DCPDA obtained from DMA.

Content of DCPDA	T g (°C)	E′ at T g + 50 °C (MPa)	ϑ e (mol·m^−3^)
0%	162.2	122.48	1.01 × 10^4^
5%	165.4	108.87	8.93 × 10^3^
10%	176.2	129.10	1.04 × 10^4^
20%	171.7	135.13	1.10 × 10^4^
30%	164.9	132.68	1.09 × 10^4^

**Table 12:** Thermal stability parameters of the 3D-printed hybrid resins with different concentrations of DCPDA obtained from TGA.

Content of DCPDA	Onset Degradation Temperature (°C)	T_10%_ (°C)	T _max_ (°C)	Char at 600 °C (%)
0%	394.7	405.8	429.6	10.56
5%	388.3	401.0	421.2	9.63
10%	394.9	405.4	425.6	10.49
20%	394.2	407.5	431.1	10.47
30%	399.3	409.0	433.6	11.19

## Data Availability

The original contributions presented in the study are included in the article/Supplementary Materials, further inquiries can be directed to the corresponding author.

## Supplementary Materials

The following supporting information can be downloaded at: https://www.mdpi.com/article/10.3390/ma19132845/s1, Table S1: Comparison of Tensile Strength and Glass Transition Temperature of Different 3D-Printed Hybrid Polymers. Table S2: Comparison of Tensile Strength and Glass Transition Temperature of Commercial 3D-Printed Resins. References [50,51,52,53] are cited in the supplementary materials.
