# Supplementary material for: Mechanical and Thermal Properties of DCPDA-Modified THEICTA/DMAA Photocurable Resins for LCD 3D Printing
Source: Materials (Basel). 2026 Jul 3;19(13):2845. doi: 10.3390/ma19132845 (PMC13362588; doi:10.3390/ma19132845)
Supplement: Supplementary file 1 [file materials-19-02845-s001.zip › materials-4366772-supplementary.pdf]

Supporting Information

Table S1. Comparison of Tensile Strength and Glass Transition Temperature of Different 3D-Printed Hybrid Polymers.

Samples	Tensile strength (Mpa)	Tg (℃)	Reference
PUA resin	14	52	[50]
Epoxy acrylate resin	60	125	[21]
Bisphenol acrylic monomers	40	57.5	[51]
Maleimide oligomer with a noncoplanar branched structure	77.1	236.1	[52]
BMI/ALCR composite resin	81.7	117.4	[23]
Isocyanate Ester Inks	80	207	[53]
CE-based IPN resin	100	240	[22]
Silica filler	73.73	123.5	[16]
This work	80.9	176.2	

Table S2. Comparison of Tensile Strength and Glass Transition Temperature of Commercial 3D-Printed Resins.

Manufacturer	Material name	Tensile strength (Mpa)	Tg (℃)
Arkama	N3D-HT511	54	148
Stratasys	P3 Defflect 110	74	143
